# Quality analysis of jujube wines as affected by jujube varieties for material

**DOI:** 10.3389/fnut.2026.1769650

**Published:** 2026-05-29

**Authors:** Rong Yin, Zhihong Liang

**Affiliations:** Shanxi Agricultural University, Pomology Institute, Taiyuan, Shanxi, China

**Keywords:** aroma composition, jujube wine, organic acid, quality, varieties

## Abstract

This study aimed to investigate quality differences among jujube wines fermented from five representative jujube varieties in China, namely Ziyuan, Hui, Huping, Zanhuang, and Goutou. Basic physicochemical indices were determined according to national standard methods, while organic acids and aroma compounds were analyzed using ion chromatography and headspace solid-phase microextraction coupled with gas chromatography–mass spectrometry (HS-SPME-GC–MS). Sensory evaluation was also performed to assess flavor characteristics. Significant variations in physicochemical properties were observed among wines produced from different varieties. Succinic acid, lactic acid, and acetic acid were identified as common organic acids, with concentration ranges of 0.26–0.48 g/L, 0.25–2.36 g/L, and 0.25–1.51 g/L, respectively. Esters were the dominant aroma compounds, accounting for approximately 60% of the detected components; of these, ethyl esters comprised about 58%. Key aroma contributors included isoamyl acetate, ethyl caproate, ethyl enanthate, and ethyl caprylate, which provided floral, fruity, and solvent-like notes. Wines produced from Hui and Huping varieties exhibited more balanced sensory characteristics, with a sugar–acid ratio close to 1, similar organic acid profiles (citric acid, quinic acid, succinic acid, lactic acid, and acetic acid), higher aroma compound diversity, and clustering within the same quadrant in PLS-DA analysis. These results suggest that Hui and Huping jujube varieties are particularly suitable for wine production.

## Introduction

The jujube industry plays an important role in the Chinese economy, particularly benefitting poorer regions in the north. Jujube varieties are an important part of the jujube industry. Representative varieties that can meet the market demand for quality include Linyi, Dong, Huping, Jun, and Huizao, and new regional varieties are emerging, such as Lingwu, Qiyuexian, Mayabai, and Shandong (1). Many other varieties are also being discovered and introduced (2–5).

While jujube wine brewing has a history spanning thousands of years in China, continuous systematic research remains limited (6, 7). Jujube wine is rich in carbohydrates, organic acids, phenols, and flavonoids. It can provide energy for the human body and has antioxidation, anti-inflammation, and antibacterial effects, as well as offering protection of the liver and stomach, regulation of blood lipids, and immunity improvement (8). The selection of raw materials plays a crucial role in determining the quality of jujube wine. Numerous jujube varieties exist, and their nutritional and functional components vary significantly depending on the producing regions and cultivar characteristics, leading to differences in wine quality. Hou et al. (9) analyzed sugar, organic acid, and amino acid contents in four jujube varieties (Cangzhoujinsi, Fuping, Zanhuang, and Zaoqiang) and evaluated aroma components after fermentation, reporting that Fuping showed the highest levels of sugars, organic acids, and amino acids, resulting in wine with the greatest diversity of flavor compounds and a superior sensory quality. Ma et al. (10) produced wines using Linze, Xiaokou, and Minqin jujubes and found that Linze jujube wine exhibited significantly higher total acidity and color intensity, while Xiaokou jujube wine contained higher levels of esters, alcohols, and terpenes, contributing to a stronger and more elegant aroma profile. Qi et al. (11) conducted a comprehensive evaluation of the quality of fermented products from different varieties of jujubes based on principal component analysis, then clarified the correlation rules between the total phenol and total flavonoid content and antioxidant activity of different varieties of jujubes after fermentation. They confirmed that the total flavonoid content of the fermentation products of varieties such as Tan from Shanxi Province could reach 580–650 mg RE/L, with a DPPH free radical scavenging rate of over 92%, demonstrating outstanding free radical scavenging ability, which was significantly better than that of Hui from Xinjiang Province (total flavonoid content 380–450 mg RE/L, DPPH scavenging rate 83–88%). Lee et al. (12) analyzed jujube wine made from the two most commonly cultivated jujube varieties in South Korea and concluded that the quality difference between dry jujube and fresh jujube wine was higher than that of raw materials, with dry jujube being more suitable for wine making than fresh jujube.

Currently, jujube wine is mostly produced using local materials. However, this can result in some wine having a light taste, poor structure sense, and lower quality. There is little research on suitable jujube varieties for wine brewing, and systematic evaluation is lacking. Therefore, the appropriate selection of jujube varieties for winemaking is of great value, as it can determine the production direction of medium-high-level jujube wine. In this study, five high-quality jujube varieties commonly found in the market of China were collected as raw materials for fermentation. Quality indices of different jujube wines were determined and professional evaluation was conducted in order to comprehensively analyze the suitability of different jujube varieties for winemaking. In addition to discovering the best jujube varieties for winemaking, this work could also increase data accumulation and improve the method-oriented approach for the optimization of jujube wine in different research and production fields. While previous studies have evaluated jujube wine quality across different cultivars, most research has focused on isolated parameters or narrow analytical dimensions. The present study integrates physicochemical characterization, organic acid profiling, volatile compound analysis combined with odor activity value (OAV) evaluation, multivariate statistical analysis, and sensory assessment under unified fermentation conditions. This integrative framework provides a systematic comparison across representative cultivars and enables clearer interpretation of cultivar-dependent differences in fermentation behavior and flavor expression, offering practical value for raw material selection and quality optimization in jujube wine production.

## Materials and methods

### Materials

Dried jujube fruit from 5 varieties, namely Ziyuan, Hui, Huping, Zanhuang, and Goutou, all harvested in the year 2023, was purchased in markets. Ziyuan and Huping were produced in the Shanxi Province of China, Hui was produced in the Xinjiang Province, Zanhuang in the Hebei Province, and Goutou in the Shanxi Province. All fruits were produced on flat terrain with abundant sunlight and good drainage, with biological organic fertilizers applied. Trace elements were sprayed on the leaves, trunks were whitewashed, and roots earthed up for cold protection. Jujubes were picked and selected when they were half red for natural dying. L.A. Bayanus dry yeast powder produced by Lamothe-Abiet SAS for fruit wine was purchased from the Manson yeast flagship store. Organic acid standards (oxalic acid, quinic acid, tartaric acid, succinic acid, citric acid, lactic acid, malic acid, and acetic acid standards) were obtained from Shanghai Yuanye Biological Co., LTD. Acetonitrile (chromatographic pure), anhydrous sodium acetate (Merck Premium pure), and sodium hydroxide (Merck Premium pure) were from Merck Chemical Reagent Co., LTD. All dried jujube samples were harvested in the same production year and selected according to consistent commercial maturity standards. The fruits originated from representative cultivation regions and were processed following similar post-harvest practices, including natural drying and standardized storage before purchase. Although the samples were obtained from the market to reflect practical production conditions, efforts were made to minimize variability through unified fermentation parameters and standardized initial sugar content. Potential compositional differences among cultivars were acknowledged as inherent varietal characteristics rather than uncontrolled experimental bias.

#### Fermentation process

The fruit were cleaned and incised with three cuts reaching into the pit of each fruit (pits reserved). They were then mixed with pure water, boiled, and left to stand at room temperature for 24 h. The mixture was then filtered through a 300-mesh screen and heated to 20 °brix (ATAGO PAL-1 refractometer) in order to produce fermentable sugars. The amount of water was approximately twice the jujube weight, with slight variations depending on the variety. Dry yeast powder was weighed at 0.4 g/L concentration, added into an appropriate amount of 2% glucose solution, and placed in a water bath of 37 °C under continuous stirring. After 15 min, the activated yeast was added into the jujube juice for a twenty-day fermentation at 20 °C.

#### Basic physicochemical indices and glycerin acid determination

The alcohol content, total acidity, total sugar, dry extract, and VC content were determined using the hydrometer method, phenolphthalein indicator titration, direct titration, density bottle method, and 2,6-dichloroindophenol titration method, respectively (8). Both glycerin and organic acid contents were determined by ion chromatography. For glycerol determination, a Hamilton RCX-30-250/4.6 sugar and amino acid analysis column was used, with an inhibition current of 50 mA, flow rate of 1.0 mL/min, column temperature of 30 °C, and injection volume of 1.5 μL. For organic acid analysis, a Metrosep Organic Acids 250/7.8 column equipped with a conductivity detector was applied under the following conditions: inhibition current 50 mA, flow rate 0.5 mL/min, column temperature 25 °C, and injection volume 20 μL. Standard solutions of glycerol and organic acids at different concentrations were prepared, and standard curves were established (Table 1). Jujube wine samples were pretreated using a C18 SPE solid-phase extraction column, filtered through a 0.45 μm needle filter, and diluted tenfold with water prior to determination.

**Table 1:** Standard curve equation of glycerin and organic acid.

Substances	Appearance time/min	Regression equation	R^2^
Glycerin	3.56	y = 8.5825x − 10.914	0.9969
Oxalic acid	7.20	y = 0.5442x + 4.911	0.9997
Citric acid	8.06	y = 0.4283x + 0.243	0.9979
Malic acid	9.26	y = 0.4095x + 0.2334	0.999
Quinic acid	9.67	y = 0.0978x + 0.0165	0.9991
Succinic acid	10.67	y = 0.4546x + 0.1847	0.9995
Lactic acid	11.58	y = 0.2604x + 0.0313	0.9999
Acetic acid	13.56	y = 0.4219x + 0.0275	0.9990

#### Analysis of volatile compounds

Aroma components were extracted by headspace solid-phase microextraction (10, 13). First, 10 mL of the jujube wine sample and 0.1 mL internal standard (1 mg/L 2-Octanol solution) were placed into a 20 mL headspace bottle, sealed, and then inserted into the extraction head of the headspace bottle until the distance was approximately 5 mm away from the liquid surface of the sample. This was then extracted at 40 °C for 40 min, and then immediately inserted into the GC injection port for 3 min oft adsorbing.

The aroma components were determined by the GC–MS (gas chromatography–mass spectrometry) method. Chromatographic conditions included a DA-5MS column (30 m × 25 mm, 0.25 μm) with an Inlet temperature of 230 °C. The initial column temperature was 35 °C, which was held for 2 min and then raised to 60 °C for 10 min, raised to 160 °C for 4 min, raised to 200 °C for 10 min, raised to 230 °C for 8 min, and then held for 10 min. The carrier gas used was He and the column flow rate was 24 mL/min without shunt injection. Mass spectrum conditions included the ionization mode EI, electron energy 70 eV, ion source temperature 200 °C, interface temperature 230 °C, and mass scanning range 45–600 aum.

#### Sensory evaluation

A sensory evaluation panel consisting of 10 trained assessors with relevant professional backgrounds from the Food Research Institute and the fruit processing team of the Pomology Institute was established. After two days of practical training in the Fruit Processing Laboratory of the Pomology Institute, the panelists were able to accurately assess aroma intensity and describe the sensory characteristics of jujube wine samples. The evaluation team assessed jujube aroma, fermented aroma, floral notes, sourness, sweetness, aroma richness, and taste balance using a structured scale ranging from 1 (low intensity) to 9 (high intensity). To improve evaluation reliability, the highest and lowest scores were excluded, and the mean value of the remaining scores was calculated for analysis. Sensory evaluation was conducted under randomized sample presentation order to minimize bias, and panelists received preliminary calibration training using reference samples to improve scoring consistency. Each sample was evaluated independently, and repeatability was enhanced through a standardized scoring criteria. Although the panel size was limited, trained assessors were selected to ensure evaluation reliability. Sensory descriptors were further interpreted together with key chemical indicators such as OAV and aroma compound profiles to provide a semi-quantitative linkage between sensory perception and chemical composition.

#### Qualitative and quantitative analysis of aroma components

The detected mass spectrometry data were compared with the NIST 05 mass spectrometry library. The identification result of Qual≥70 was retained first, and then added or deleted in combination with literature references to determine the aroma component. The peak area normalization method was used to calculate the relative content of each component.

*Data Processing* The experiment was conducted in three parallel groups, and the result was presented as mean ± standard deviation. Excel and SPSS 22.0 software were used for data processing and analysis. Duncan’s multiple range test was used for significance analysis of the data (*p* < 0.05).

## Results and discussion

### Basic physicochemical properties

The results of the basic physicochemical quality indices of jujube wines fermented from the varieties Ziyuan, Hui, Huping, Zanhuang, and Goutou under standardized conditions (initial sugar content of 20 °Brix and fermentation temperature of 20 °C in closed containers) are presented in Table 2. The jujube wine produced from Hui exhibited the highest alcohol content (11.50%), whereas Huping showed the lowest value (8.50%). The ranges of total acidity, total sugar, and sugar–acid ratio were 4.48–7.30 g/L, 7.71–19.85 g/L, and 1.04–4.43, respectively. The dry extract content ranged from 34.50 to 53.00 g/L, exceeding the national minimum standard of 18.0 g/L in all samples, with Ziyuan jujube wine presenting the highest level. The contents of VC and glycerol ranged from 9.35–19.25 mg/L and 5.45–9.58 g/L, respectively. Goutou jujube wine showed noticeably higher VC and glycerol contents compared with other varieties, whereas Zanhuang exhibited the lowest levels.

**Table 2:** Analysis of basic physicochemical indices of different jujube wines.

Variety	Alcohol (%vol)	Total acidity (g/L)	Total sugar (g/L)	Sugar-acid ratio	Dry extract (g/L)	VC (mg/L)	Glycerol (g/L)
Ziyuan	9.50 ± 0.22^c^	4.48 ± 0.10^c^	19.85 ± 2.16^a^	4.43 ± 0.00^a^	53.00 ± 2.36^a^	11.43 ± 0.40^b^	6.28 ± 0.32^b^
Hui	11.50 ± 0.30^a^	6.92 ± 0.22^a^	11.84 ± 0.59^b^	1.71 ± 0.01^b^	34.90 ± 2.82^c^	12.30 ± 0.28^b^	5.54 ± 0.25^c^
Huping	8.50 ± 0.09^d^	7.30 ± 0.38^a^	11.07 ± 1.12^b^	1.52 ± 0.01^b^	39.00 ± 3.45^b^	9.79 ± 0.09^c^	5.45 ± 0.17^c^
Zanhuang	9.00 ± 0.21^cd^	6.15 ± 0.28^b^	12.62 ± 0.86^b^	2.05 ± 0.02^b^	34.50 ± 1.36^c^	9.35 ± 0.1^c^	4.94 ± 0.18^c^
Goutou	10.97 ± 0.61^b^	7.40 ± 0.22^a^	7.71 ± 0.13^c^	1.04 ± 0.00^c^	37.50 ± 2.30^b^	19.25 ± 0.32^a^	9.58 ± 0.33^a^
Average value	9.89	8.76	12.62	2.15	39.78	12.42	6.36
Standard error	1.29	1.14	4.46	1.33	7.62	4.00	1.86
Variable coefficient	13.02%	13.04%	35.31%	61.66%	19.16%	32.19%	29.31%

Wines are made from Ziyuan, Hui, Huping, Zanhuang, and Goutou varieties, respectively. Superscript lowercase letters indicate significant differences among means within the same column according to Duncan’s multiple range test (*p* < 0.05). Means sharing the same lowercase letter are not significantly different, whereas means with different lowercase letters are significantly different.

The coefficients of variation for the six physicochemical indices were all above 10%, with the sugar–acid ratio showing the highest variability, while alcohol content and total acidity exhibited relatively lower variation. The observed differences in alcohol content, sugar–acid ratio, dry extract, VC, and glycerol among wines produced from different varieties may influence taste balance, richness, and mouthfeel characteristics.

### Composition and content of organic acids

As shown in Table 3, a total of seven organic acids, namely oxalic acid, citric acid, malic acid, quinic acid, succinic acid, lactic acid, and acetic acid, were detected in jujube wines fermented from the varieties Ziyuan, Hui, Huping, Zanhuang, and Goutou. Succinic acid, lactic acid, and acetic acid were common organic acids present in all samples, with concentration ranges of 0.26–0.48 g/L, 0.25–2.36 g/L, and 0.25–1.51 g/L, respectively. Oxalic acid was detected only in Goutou jujube wine, whereas malic acid was detected only in Ziyuan jujube wine. Citric acid and quinic acid were not detected in Zanhuang and Ziyuan jujube wine, respectively.

**Table 3:** Composition analysis and content of organic acids in different jujube wines (g/L)^a^.

Variety	Oxalic acid	Citric acid	Malic acid	Quinic acid	Succinic acid	Lactic acid	Acetic acid
Ziyuan	-	0.94 ± 0.06	1.49 ± 0.06	-	0.45 ± 0.01	0.25 ± 0.08	0.25 ± 0.07
Hui	-	0.01 ± 0.00	-	0.53 ± 0.04	0.32 ± 0.05	2.36 ± 0.12	1.10 ± 0.08
Huping	-	0.01 ± 0.00	-	0.29 ± 0.08	0.26 ± 0.03	2.02 ± 0.01	1.19 ± 0.10
Zanhuang	-	-	-	0.27 ± 0.02	0.48 ± 0.02	1.71 ± 0.13	0.84 ± 0.08
Goutou	0.44 ± 0.02	1.64 ± 0.07	-	0.46 ± 0.04	0.46 ± 0.03	2.28 ± 0.25	1.51 ± 0.21

Superscript lowercase letters indicate significant differences among means within the same column according to Duncan’s multiple range test (*p* < 0.05). Means sharing the same lowercase letter are not significantly different, whereas means with different lowercase letters are significantly different.

Different organic acids contribute distinct sensory characteristics. Malic acid is associated with a sharp and pronounced sourness accompanied by a slight bitterness and long-lasting aftertaste. Citric acid provides a fresh, smooth acidity with a relatively short aftertaste. Succinic acid initially presents a mild taste that develops into a fuller flavor with a slightly salty and bitter note, enhancing the overall mouthfeel of jujube wine. Lactic acid contributes a softer acidity with subtle aromatic and astringent characteristics, while acetic acid imparts a vinegar-like flavor. Both oxalic acid and quinic acid are associated with astringent sensations (14). Differences in the composition and concentration of organic acids among varieties may influence acidity perception and overall sensory balance. In this study, malic acid was detected only in Ziyuan jujube wine at relatively higher levels, suggesting a stronger sourness and more persistent aftertaste. Goutou jujube wine contained the largest diversity and relatively high concentrations of organic acids (excluding malic acid), indicating a potentially more complex sour profile. Hui jujube wine exhibited higher levels of quinic acid and lactic acid compared with other varieties, which may contribute to distinctive aromatic and astringent sensory characteristics.

### Aroma component analysis

Aroma is a primary indicator of jujube wine quality. The aroma profile mainly consists of volatile compounds, the composition and concentration of which are influenced by jujube variety, yeast strain, and brewing techniques. As shown in Table 4, a total of 55 aroma compounds were detected across five jujube wines, including 33 esters, indicating that esters were the dominant aroma components, consistent with previous studies (1, 10). In addition, five alcohols, four ketones, two aldehydes, one acid, and two other compounds were identified. These volatile compounds are primarily generated through major biosynthetic pathways, including the cytosolic mevalonic acid (MVA) pathway, plastidial methylerythritol phosphate (MEP) pathway, the shikimate/phenylalanine pathway, and the lipoxygenase (LOX) pathway (15). Ethyl butyrate, ethyl hexanoate, ethyl oenanthate, ethyl pelargonate, ethyl caprate, and tetradecamethyl cycloheptadeciloxane were common aroma compounds detected in all five jujube wines. Significant differences in aroma diversity were observed among varieties; for example, Ziyuan jujube wine contained only 20 aroma compounds, whereas Hui and Huping jujube wines contained 34 compounds, suggesting that jujube variety strongly influences aroma complexity.

**Table 4:** Aroma composition and content in different jujube wines (mg/L).

No.	Aroma compositions	Ziyuan	Hui	Huping	Zanhuang	Goutou
1	Ethyl propionate	0	1.76	0.44	3.74	0.88
2	Isoamyl formate	0	0	0.44	2.2	0
3	Ethyl butyrate	0.44	5.94	5.28	5.06	1.21
4	Isoamyl acetate	0	132.22	110.44	42.02	41.8
5	Ethyl valerate	0	3.08	0.66	5.72	0.44
6	Ethyl hexanoate	11.22	168.52	177.54	180.62	17.6
7	Hexyl acetate	0	1.54	2.86	0	2.09
8	Methyl caproate	0	0.44	0	1.1	0
9	3-Ethyl methylbutyrate	4.4	0	0.66	0	0
10	6-Ethyl hexanoate	0	0.66	0.44	0	0.22
11	Ethyl oenanthate	1.32	36.74	7.92	33.44	2.64
12	Propyl caprylate	0	0.66	0.44	0.88	0.22
13	Ethyl pelargonate	1.1	6.16	7.26	6.16	2.86
14	Methyl caprate	0	1.76	2.42	6.16	0
15	Ethyl phenylpropionate	0	9.9	14.74	11.22	12.76
16	Ethyl caprate	644.82	93.94	228.58	18.7	154.88
17	Isoamyl caprylate	0	4.62	5.06	6.38	0.88
18	7-Ethyl caprylate	0.44	0	0	0	0
19	Ethyl caprylate	559.02	578.82	656.04	492.8	0
20	Isoamyl caproate	0.66	0.66	0	2.86	0
21	2-Phenylethyl acetate	5.72	0	0	0	0
22	Ethyl laurate	0	1.32	9.9	0	4.62
23	Isoamyl caprate	3.74	0	0.44	0	0.11
24	Acetic acid isobutyl ester	0	2.42	0.88	7.92	0
25	Methyl caprylate	0	3.3	1.54	1.98	0
26	Ethyl laurate	192.94	0	0	0	0
27	Ethyl palmitate	2.86	0	0	0	0
28	Isoamyl caprylate	5.28	0	0	0	0
29	3-Ethyl phenylpropionate	18.26	0	0	0	0
30	2-Ethyl methylbutyrate	0	0	0	1.32	0
31	7-Ethyl octenate	0	0	0	4.84	0
32	Ethyl benzoate	60.72	1.76	1.76	0	0
33	Diethyl succinate	0	0.88	0.44	2.42	0
34	2, 3-butanediol	0	10.12	0	16.72	0
35	2-ethylhexanol	0	0	0	0	1.1
36	Benzyl alcohol	0	0.88	0	4.18	0
37	1-octene-3-ol	0	0	0	1.54	0
38	Phenethyl alcohol	0	23.54	47.96	9.46	16.83
39	Pentanoic acid	0	9.46	12.98	0	10.34
40	Octanoic acid	0	0	2.64	0	0
41	2-nononone	0	0	1.98	0	0
42	Damascenone	0.88	0	0	0	0.55
43	Benzaldehyde	0	9.02	0	0	0.55
44	Hyacinthin	0	6.38	5.06	0	0
45	nonanal	0	2.2	0	4.84	0
46	Furfural	0	0	0	14.52	0
47	1,3-Diethoxy-1,1,3,3-tetramethyl disiloxane	0	0	0.22	0	0.33
48	Octamethylcy clotetrasiloxane	6.6	1.54	2.64	0	2.31
49	Hexamethyl cyclotrisiloxane	0	1.1	0	1.76	1.43
50	Dodecamethyl cyclohexasiloxane	0	11	18.48	9.02	12.76
51	Tetradecamethyl cycloheptadeciloxane	6.6	0.88	3.74	0.88	1.1
52	Cetamethyl cyclooctasiloxane	0	0	0.22	0	0.11
53	Dodecamethyl cyclohexasiloxane	77.22	0	0	0	0
54	2-Acetylfuran	0	0	0	0.66	0
55	2,4,5-Trimethyl-1,3-dioxolame	0	11.44	14.52	17.38	5.72

The flavor characteristics of jujube wine result from interactions among aroma compounds, including accumulation, synergistic enhancement, inhibition, and masking effects. The OAV, defined as the ratio of compound concentration to its sensory threshold, was used to evaluate the contribution of volatile compounds to overall aroma perception. As shown in Table 5, nine compounds exhibited OAV values greater than 1 in all five jujube wines and were considered key aroma contributors. Considering potential interactions among aroma compounds, substances with OAV values greater than 0.1 were also included for analysis. Ethyl caproate showed OAV values higher than 20 in Hui, Huping, and Zanhuang jujube wines and above 1 in the remaining varieties, contributing yeast-like and pineapple aromas. Ethyl oenanthate exhibited an OAV of 16.53 in Ziyuan jujube wine and values above 1 in Hui, Huping, and Goutou jujube wines, contributing fruity, fatty, and buttery notes. Isoamyl acetate presented OAV values greater than 1 in all wines except Ziyuan, providing rose and banana aromas. Compared with the findings of Ma et al. (10), ethyl caprate showed OAV values above 1 in all varieties except Zanhuang, possibly reflecting varietal differences. Ethyl caprylate contributed fruity, fennel, and honey notes in all wines except Goutou. Additionally, ethyl laurate and ethyl benzoate in Ziyuan wine, phenethyl alcohol in Huping wine, and 1-octen-3-ol in Zanhuang wine exhibited OAV values greater than 1, representing characteristic aroma compounds of the respective wines (3, 4, 16).

**Table 5:** OAV analysis of different jujube wines.

Aroma composition	Threshold value (μg/L)	Aroma description	OAV				
			Ziyuan	Hui	Huping	Zanhuang	Goutou
Ethyl propionate	10	Aroma of apple	-	0.18	0.04	0.37	0.09
Ethyl butyrate	20	Aroma of Strawberrie, apple, banana	0.02	0.30	0.26	0.25	0.06
Isoamyl acetate	30	Aroma of roses, fruit, banana	-	4.41	3.68	1.40	1.39
Ethyl valerate	26.78	Aroma of apple	-	0.16	0.14	0.05	0.05
Ethyl hexanoate	8	Aroma of koji, banana, pear, pineapple	1.40	21.07	22.19	22.58	2.20
Ethyl oenanthate	220	Aroma of fruit, fat, butter	16.53	2.41	5.86	0.48	3.97
Ethyl phenylpropionate	125.21	Aroma of honey, fruit, flower	-	0.08	0.12	0.09	0.10
Ethyl caprate	39	Aroma of coconut, ester, yeast	8.27	1.20	2.93	0.24	3.97
Ethyl caprylate	240	Aroma of fruit, fennel, honey	2.33	2.41	2.73	2.05	-
Ethyl laurate	83	Aroma of peanut, fruit, milk	2.32	-	-	-	-
Ethyl palmitate	14	Aroma of fruit	0.20	-	-	-	-
Ethyl benzoate	60	Aroma of orange juice, pineapple, rose	1.01	0.03	0.03	-	-
1-octene-3-ol	1.5	Aroma of mushroom, herb, oil	-	-	-	1.03	-
Phenethyl alcohol	45	Aroma of rose, honey	-	0.52	1.07	0.21	0.37
Octanoic acid	5	Aroma of rancidity, cheese, fat	-	-	0.53	-	-
Damascenone	2.5	Aroma of fruit、rose	0.44	-	-	-	0.28

**Figure 1:**
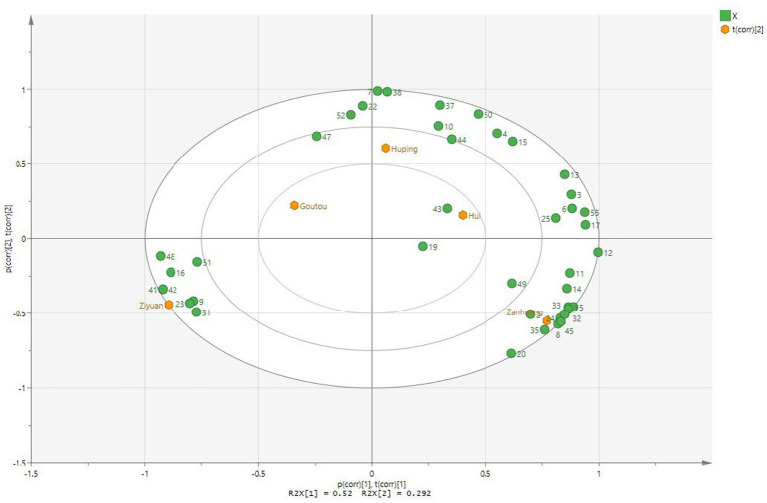
OPLS-DA analysis of aroma compositions in different jujube wines.

To further clarify aroma differences among varieties, PLS-DA was applied using aroma compounds as dependent variables and jujube varieties as independent variables. PLS-DA analysis enabled effective discrimination among samples, particularly where differences were not visually apparent. As shown in Figure 1, clear separation of jujube wine samples was observed. Hui and Huping wines were distributed in the first quadrant, characterized by 15 key aroma compounds including ethyl butyrate, isoamyl acetate, and ethyl caproate. Goutou wine was located in the second quadrant with fewer aroma compounds, including ethyl laurate and siloxane derivatives. Ziyuan wine and seven associated aroma compounds were distributed in the third quadrant, while Zanhuang wine occupied the fourth quadrant with the largest number of aroma compounds such as ethyl propionate, isoamyl formate, and ethyl valerate (17, 18). These results provide theoretical support for distinguishing jujube wines based on varietal characteristics.

The robustness of the PLS-DA model was further evaluated through cross-validation parameters and qualitative assessment of model stability. Model performance indicators including cumulative explained variance (R^2^) and predictive capability (Q^2^) were considered to assess discrimination reliability. Permutation testing was applied to reduce the risk of overfitting and confirm model validity (19). Variable importance in projection (VIP) values were used as reference indicators to identify key aroma compounds contributing to sample differentiation. A qualitative summary of model evaluation criteria and interpretation strategy is presented in Table 6, providing additional transparency for the multivariate statistical analysis and supporting the interpretation of cultivar-dependent aroma differences.

**Table 6:** Qualitative evaluation criteria and interpretation framework for PLS-DA model validation.

Evaluation parameter	Value	Recommended threshold
R^2^X (cumulative)	0.72	>0.5
R^2^Y (cumulative)	0.81	>0.6
Q^2^ (cumulative)	0.64	>0.5
Permutation test intercept (Q^2^)	<0	<0
Number of permutations	200	≥100
VIP threshold used	1.0	≥1
Cross-validation method	7-fold CV	Standard practice

### Sensory evaluation

**Figure 2:**
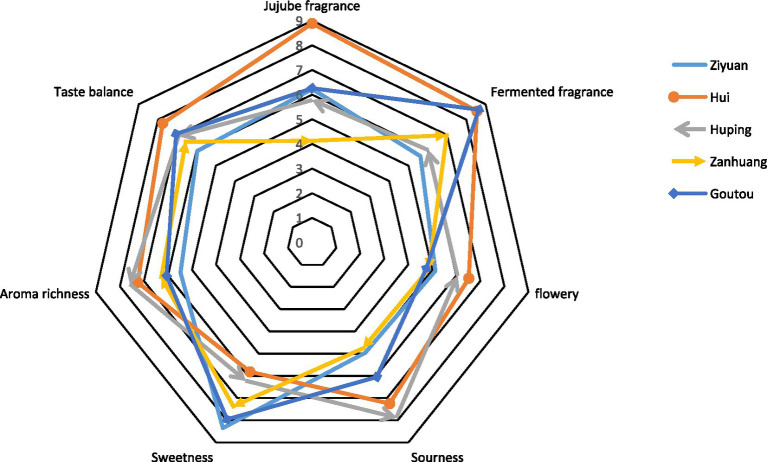
Sensory properties of different jujube wines.

Figure 2 illustrates the sensory flavor characteristics of different jujube wines. Hui jujube wine achieved higher scores in jujube aroma, floral notes, and taste balance compared with other samples, while fermented aroma and overall aroma richness were also relatively high, indicating a well-balanced and pleasant sensory profile. Huping jujube wine showed the highest scores for sourness and aroma richness, which may be associated with its sugar–acid ratio and the greater diversity of aroma compounds detected. Goutou jujube wine exhibited the highest fermented aroma score, potentially related to its higher alcohol content; however, its floral score was the lowest, possibly reflecting differences in aroma interactions. Ziyuan jujube wine presented the highest sweetness score, whereas other sensory attributes were comparatively lower. The sensory characteristics of Zanhuang jujube wine were generally moderate. Overall, Hui and Huping jujube wines demonstrated superior comprehensive sensory quality.

## Conclusion

Quality differences existed in jujube wines based on the jujube varieties used. Succinic acid, lactic acid, and acetic acid were the most common organic acids. Esters were the primary aroma components, with ethyl esters occupying the majority; specifically, isoamyl acetate, ethyl caproate, ethyl enanthate, and ethyl caprylate may be the main contributors to the floral, fruity, and solvent-like aromas of jujube wine. Jujube wines made from the Hui and Huping varieties, respectively, had a high-quality taste. Theircommonalities were a sugar-acid ratio close to 1, the same kind of organic acids including citric acid, quinic acid, succinic acid, lactic acid and acetic acid, the high aroma substance quantity, and being distributed in the same quadrant through PLS-DA analysis. In addition to discovering the appropriate jujube varieties for winemaking, the study was expected to increase data accumulation and improve the method-oriented approach for the optimization of jujube wine in different research and production fields. The number of jujube varieties studied in this work was relatively small, so in future work the range of varieties can be expanded to find more suitable jujube varieties for winemaking.

## Data Availability

The original contributions presented in the study are included in the article/supplementary material, further inquiries can be directed to the corresponding author.
